# Neonatal spectral EEG is prognostic of cognitive abilities at school age in premature infants without overt brain damage

**DOI:** 10.1007/s00431-020-03818-x

**Published:** 2020-09-29

**Authors:** Elisa Cainelli, Luca Vedovelli, Isabella Lucia Chiara Mariani Wigley, Patrizia Silvia Bisiacchi, Agnese Suppiej

**Affiliations:** 1 Department of General Psychology, University of Padova, via Venezia 8, 35131 Padova, Italy; 2 Child Neurology and Clinical Neurophysiology, Padua University Hospital, via Giustiniani 3, 35133 Padova, Italy; 3 Lab LeSexp, Unit of Biostatistics, Epidemiology, and Public Health, Department of Cardiac, Thoracic, Vascular Sciences, and Public Health, University of Padova, via Loredan 18, 35131 Padova, Italy; 4 Department of Developmental and Social Psychology, University of Padova, via Venezia 8, 35131 Padova, Italy; 5 Padova Neuroscience Centre, PNC, Padova, Italy; 6 Department of Medical Sciences, Pediatric Section, University of Ferrara, via Aldo Moro 8, 44124 Cona, Fe Italy

**Keywords:** Neonate, Reticular activating system, Arousal–attentional system, Quantitative EEG, Long-term outcome, Preterm birth

## Abstract

Prematurity is a prototype of biological risk that could affect the late neurocognitive outcome; however, the condition itself remains a non-specific marker. This longitudinal 6-year study aimed to evaluate the prognostic role of neonatal spectral EEG in premature infants without neurological complications. The study cohort was 26 children born 23–34 gestational ages; all neonates underwent multichannel EEG recordings at 35 weeks post-conception. EEG data were transformed into the frequency domain and divided into delta (0.5–4 Hz), theta (5–7 Hz), alpha (8–13 Hz), and beta (14–20 Hz) frequency bands. At 6 years, a neuropsychological and behavioral evaluation was performed. Correlations between spectral bands and neuropsychological assessments were performed with a conservative and robust Bayesian correlation model using weakly informative priors. The correlation of neuropsychological tasks to spectral frequency bands highlighted a significant association with visual and auditory attention tests. The performance on the same tests appears to be mainly impaired.

*Conclusions*: We found that spectral EEG frequencies are independent predictors of performance in attention tasks. We hypothesized that spectral EEG might reflect early circuitries’ imbalance in the reticular ascending system and cumulative effect on ongoing development, pointing to the importance of early prognostic instruments.

**Table:** 

**What is Known:** *• Prematurity is a non-specific marker of late neurocognitive risk.* *• Precise prognostic instruments are lacking, mostly in patients with low-grade conditions.* **What is New:** *• Longitudinal long-term studies are scarce but crucial for the inferential attributive process.* *• Spectral EEG frequencies are independent predictors of performance in attention tasks.*

**Electronic supplementary material:**

The online version of this article (10.1007/s00431-020-03818-x) contains supplementary material, which is available to authorized users.

## Introduction

Long-term neurodevelopmental impairments remain a major concern after premature birth, particularly in infants born at the lowest gestational ages [1–3]. Prematurity is a prototype of biological risk that could affect the neurocognitive outcome; however, it remains a non-specific marker [4]. It may negatively affect the normal maturational processes also in infants without overt brain damage or medical complications [5, 6]. Thus, even apparently healthy children, who did not develop major sequelae in the first years of life, are at risk for neurocognitive impairments emerging at older ages, typically during the school period [7, 8]. Deficits are reported in visual-motor [9, 10], linguistic [11], attention and executive functions [12], and learning and achievements [13–15]. Furthermore, children may manifest behavioral and psychological problems [16].

Abnormalities in high-order neuropsychological functions require many years to manifest [17–19], due to the slow rate of maturation of complex abilities such as attention and executive functions. Abnormalities in circuitry formation begin early but will manifest only when the system is no longer able to compensate for the constantly increasing demands of the surrounding environment. The first years of age are characterized by a high plasticity, but, once consolidated, the altered pattern of functioning may become a stable characteristic, as shown by studies on long-term follow-up in the adolescence and adulthood [16, 20].

Although not so disabling compared with cerebral palsy and intellectual disability, long-term neuropsychological and behavioral impairments affect the life quality of children and their families. Their incidence is growing [21], and school and sanitary services are increasingly overloaded. The early identification of children at risk is hampered by the scarcity of good neonatal markers, mainly when the perinatal period runs without medical complications or signs of brain damage.

Several functional neuroimaging studies highlighted abnormalities in premature brain functioning even in the absence of overt brain damage [1, 22–24]. The electrophysiological tools have the advantage over imaging techniques of being less expensive and available at the bedside. Abnormal developmental trajectories of early prematurity could be detected as early as 35 weeks post-conception, both using event-related potentials [25–27] and quantitative EEG [28–30].

Power spectral analysis is a simple, objective, and sensitive method for quantifying the digitized EEG.

The prognostic value of spectral EEG analysis on long-term sequelae is yet scarcely investigated. Still, the few available literature data suggest good prognostic abilities [31], also in children born prematurely [32]. Long-term longitudinal studies are crucial in developmental cognitive neuroscience, for the inferential attributive process and in the understanding of early developmental trajectories. Their use is limited by the need for covering the years elapsing from the neonatal period to the age when complex cognitive functions develop and can be tested.

This prospective longitudinal 6-year study aimed to evaluate the prognostic role of spectral EEG recorded at 35 weeks post-conception in premature infants free of medical and neurological complications, attaining school age. Thirty-five-week gestation is a critical time of brain maturation [33], and neurophysiological testing close to this period, rather than 40 post-conception, might highlight subtle and/or transient abnormalities before the compensation mechanisms occur, and could have a role in long-term prognosis.

## Methods

### Participants

The study cohort was a subset of 26 children born between January 2011 and January 2012, recruited from our ongoing prospective study on perinatal risk factors and long-term outcomes of neonates admitted to the neonatal intensive care unit.

Inclusion criteria for the present study were gestational age at birth lower than 35 weeks, having successfully performed a neonatal multichannel EEG of at least 1-h duration at a corrected age of 35 weeks, written consent of the parents to the study, and adherence to all the follow-up.

Exclusion criteria were neonatal neurological risk factors as detailed elsewhere [28]. Briefly, neonates were recruited when none of the following neurological risk factors was present: intrauterine growth restriction (defined as an estimated fetal weight below the 10th percentile and umbilical artery pulsatility index greater than 2 standard deviations), craniofacial malformations, clinical evidence of neonatal encephalopathy, brain ultrasound evidence of intra-ventricular hemorrhage or periventricular cystic leukomalacia, occurrence of seizures, treatment with drugs (e.g., sedatives) affecting the central nervous system. Furthermore, we excluded children with abnormal EEG traces as evaluated by visual inspection, a post-neonatal diagnosis of genetic, metabolic or neurodegenerative syndrome or intellectual disability, cerebral palsy, sensorial invalidating deficits, epilepsy, at any time during the follow-up period. Seventeen patients out of the total cohort of 26 patients were part of a previous study reporting the outcome at 1 year of age [28].

The patient’s clinical characteristics are reported in Table 1.

**Table 1:** Clinical data of the children recruited for the study

GA mean (range)	29.5 (23–34)
Birth weight (g)	1350 ± 391
Birth length (cm)	37.6 ± 4.4
Birth CC (cm)	28.4 ± 3.5
Male rate	16 (61.5%)
1°-min Apgar score	6.8 ± 1.6
5°-min Apgar score	8.2 ± 0.97
pH at birth	7.25 ± 0.12

*GA*, gestational age; *PCA*, post-conceptional age; *CC*, cranial circumference

All procedures contributing to this work comply with the ethical standards of the relevant national and institutional committees on human experimentation and with the Helsinki Declaration of 1975, as revised in 2008. The Institutional Ethical Committee approved the study (Comitato Etico per la Sperimentazione Clinica dell’Azienda Ospedaliera di Padova, Prot. N. 1693P).

### Neonatal neurophysiological assessment

Recordings were performed before discharge from the hospital when infants were clinically stable. Post-conceptional age was computed as the sum of gestational age at birth, and the period of extra-uterine life elapsed from birth to the day of EEG recording [34].

The methodology for EEG recording was previously detailed [28].

In brief, electrodes were placed according to the 10–20 International System of electrode placement and international guidelines for neonates. We used a Galileo EEG system (EB Neuro, Florence, Italy). We choose to analyze EEG segments recorded in active sleep because this state represents predominantly neocortical activity [35]. We considered for spectral analysis, only EEG segments where both EEG and behavioral evaluation confirmed an active sleep stage. For offline analysis, 30 min of artifact-free EEG traces were selected from at least 1-h recording.

### Data analysis

Pre-processing and spectral analysis were performed as previously described [28] using the EEGLAB toolbox and a custom-scripted software in the MATLAB environment. In brief, the frequency spectrum was divided into the following bands: delta (0.5–4 Hz), theta (5–7 Hz), alpha (8–13 Hz), beta (14–20 Hz). Absolute power (defined as the integral of all powers within the frequency band, expressed in μV^2^) was calculated from the transformed signal. As the total absolute spectral power may vary considerably, spectral values among subjects were normalized for total power and expressed as relative spectral power measures (defined as the ratio of absolute band power and total power of all bands, expressed in percentage).

Finally, we calculated the main total spectral power for the delta, theta, alpha, and beta bands by performing the mean of Fz, C3, Cz, C4, T3, and T4 locations activity. The Fp1, Fp2, O1, and O2 locations were excluded because of the numerous artifacts on these channels.

**Fig. 1:**
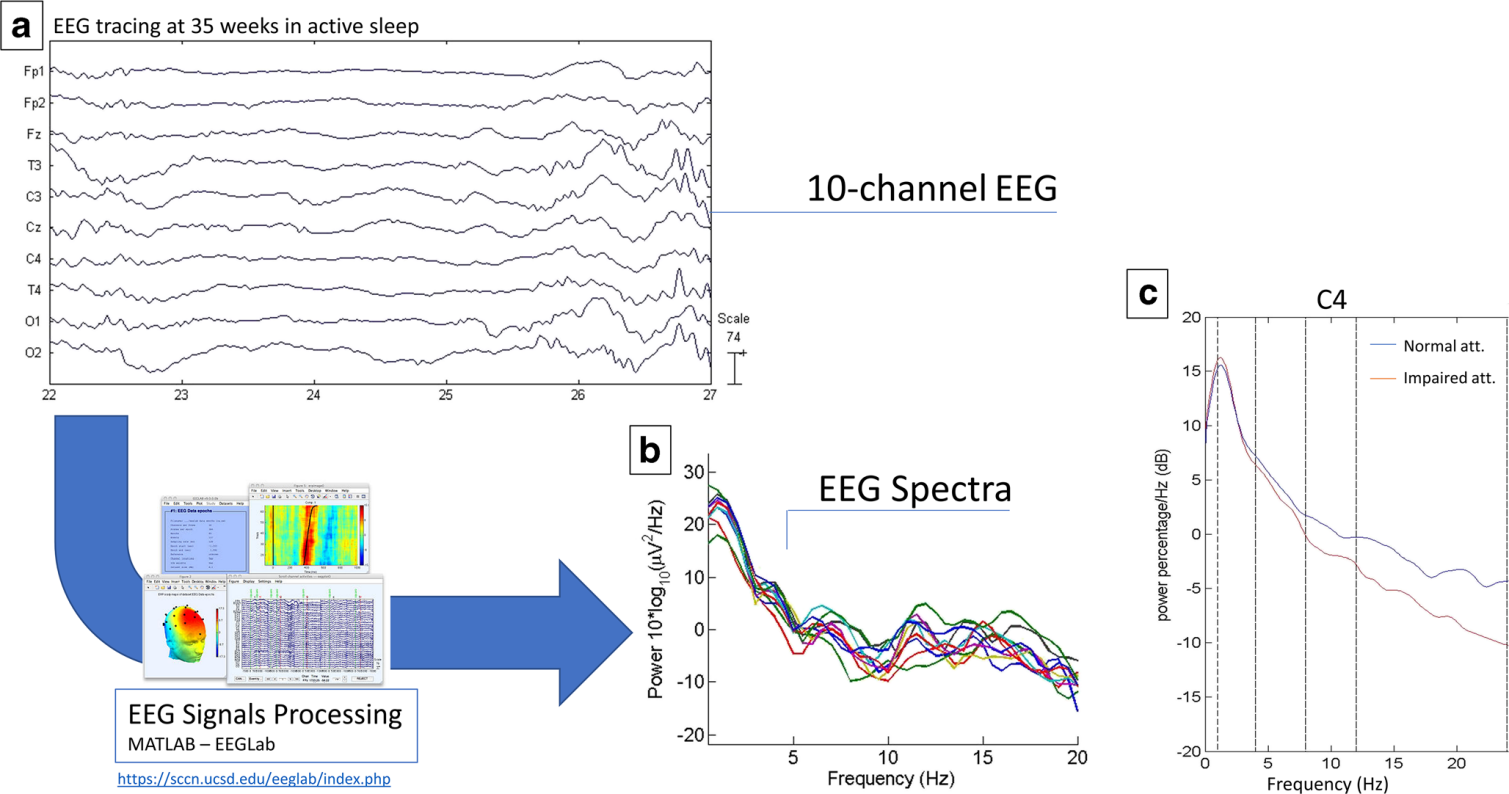
The processing procedure of transformation the EEG tracing (panel A) of one neonate at 35 weeks post-conception in active sleep in the frequency spectrum (panel B). Panel C shows the EEG power spectra at 35 post-conception on C3 channel of children with impaired and normal performance to the visual attention task

An example of the processing of EEG tracing in the frequency spectrum is reported in Fig. 1 (panels A and B).

### Follow-up neuropsychological assessment

Neuropsychological assessment was conducted by a psychologist trained in test administration and scoring (E.C.) at the mean age of 6 years (SD 0.45).

#### Cognitive assessment

We used the Wechsler Preschool and Primary Scale of Intelligence III (WPPSI-III) test or the Wechsler Intelligence Scale for Children IV (WISC-IV), standardized for Italian sample, to evaluate general cognitive performance [36–38]. All results were expressed as an age-standardized score, with a population means of 100 and a standard deviation of 15.

#### Neuropsychological testing

The following cognitive domains were assessed: language, using the naming test and the semantic verbal fluency test, which evaluates the ability to access the lexicon through a categorial cue [39]; attention, using the visual and auditory attention tests of the NEPSY-II [40, 41]; memory, using the Corsi block-tapping test, which evaluates short-term verbal and visuo-spatial memory, and the word’s list and list recall, which evaluate learning and long-term verbal memory [39]; executive functions, using the Tower of London test, which evaluates planning and problem-solving [42], the Coding test of the WISC-IV or WPPSI-III [37, 38], the Stroop Test, which evaluates inhibitory control [43]; visuo-motor functions, using the visual-motor integration (VMI, [44]); social skills, using the Theory of Mind and the Emotional recognition tests of the NEPSY-II [40, 41]. The entire test battery required nearly 3 h, divided into two meetings, to be completed.

We administered to the parents the questionnaire Conners’ Rating Scales-Revised (CRS-R) in order to identify the presence of behavioral problems and ADHD. The Conners Parent Rating Scales-long version (CPRS-R:L) report parent ratings of child behaviors involving problems in seven psychopathological areas: oppositional, inattention, hyperactive, anxious–shy, perfectionism, social problems, and psycho-somatic. For the analysis, total scores were considered: the ADHD total score, the CGI (Conners global index) total score, and DSM-IV total score [45].

### Statistical analysis

Continuous variables were tested for normality and summarized as mean and standard scores. Scores for the cognitive, neuropsychological, and questionnaires were age-corrected and converted into *z* scores and scaled scores (neuropsychological tests), *T* scores (questionnaires), or standard scores (cognitive tests), based on published normative data. The *z* scores indicate the deviation from the mean population score, which is set to 0, standard deviation 1. A *z* score of − 2 (or less) comprised 2.5% of the normal distribution and was considered to be significantly lower than average. Scaled scores indicate the deviation from the mean population score, which was set to 10, standard deviation 3. A scaled score of 4 (or less) was considered to be significantly lower than average. The *T* scores indicate the deviation from the mean population score, which was set to 50, standard deviation 10. A *T* score of 70 (or more) indicates a clinical condition.

Results of the WPPSI-III and of the WISC-IV have been converted in standard scores having mean 100 and standard deviation 15. Impairment was defined as a standard score lying two standard deviations below the mean (< 70).

We evaluated the direct correlations between neuropsychological and EEG spectral data using a regression model robust to outliers with bivariate Student’s *t* distribution [46]. We performed the analysis in a Bayesian framework to avoid arbitrary multiple-comparisons corrections and to minimize false discovery rates by imposing weakly informative priors on the model’s parameters [47, 48]. Specifically, we used LKJ prior distribution [49] on the correlation’s parameters with the concentration parameter equal to 4. Such prior distribution, highly concentrated around zero, minimizes the risk of observing non-null correlations, which may arise only by random chance, as it often occurs in small sample size settings, like in our study. Significant correlations were identified as those whose posterior intervals do not contain the zero value, i.e., the value of no correlation. The sampling from the posterior distribution of the model’s parameters was carried out using the Hamiltonian Monte Carlo (HMC) algorithm with Stan software for Bayesian inference [50]. The algorithm was run with four chains and 2000 iterations, of which 500 were discarded as warm-up. The convergence of the algorithm was assessed using trace plots and an improved version of the R-hat [51].

We implemented the statistical analysis in R software for statistical computing [52] (version 3.6.2). The *brms* package was used to fit the models (version 2.11.1) [53]. The full R code of the model is available in the supplementary materials.

## Results

Mean scores were in the range of normality for all the cognitive and neuropsychological domains explored (Table 2).

**Table 2:** Mean and standard deviation scores of cognitive and neuropsychological tasks

Domain	Test	Mean ± SD
General intelligence	IQ	96.6 ± 20.5
Language	Semantic fluency (*z* scores)	− 0.86 ± 0.56
	Naming (*z* scores)	− 0.21 ± 0.48
Memory	Corsi (*z* scores)	0.14 ± 0.68
	Word’s list (*z* scores)	0.26 ± 0.91
	List recall (*z* scores)	0.09 ± 2.47
Visual-motor abilities	VMI (SS)	10.8 ± 0.0
Executive functions	TOL (*z* scores)	− 0.42 ± 0.12
	Stroop (*n*, % impaired)	2 (7%)
	Coding (SS)	7.64 ± 6.36
Attention	Visual attention (SS)	10.8 ± 1.41
	Auditory attention (*n*, % impaired)	7 (27%)
Social skills	Mind’s theory total (SS)	8.45 ± 2.82
	Emotional recognition (SS)	7.65 ± 5.65

*SD*, standard deviation; *IQ*, intelligence quotient; *VMI*, visual-motor integration test; *SS*, scaled scores; *TOL*, Tower of London

The Bayesian correlation model converges for all the analyzed pairs of variables.

By considering individual impairments in cognitive and neuropsychological tests, six children exhibited a borderline cognitive profile (70 > IQ < 85); eight showed at least two neuropsychological impaired tests (< 2 standard deviations). By considering individual impairments on total scores of CPRS-L questionnaire, five children obtained a borderline score in the ADHD total score, one child a borderline score in CGI total score, and finally, four impaired sores in the DSM-IV total score.

The correlation of neuropsychological tasks to spectral frequency bands highlighted a significant association with visual and auditory attention tests (Table 3). Figure 1 (panel C) shows the EEG power spectra at 35 post-conception on C3 channel of children with impaired and normal performance to the visual attention task.

**Table 3:** Non-zero correlations from the Bayesian model

Parameters	Correlation coeff. Median (95% C.I.)
Visual attention vs. TOT alpha	0.46 (0.10–0.71)
Visual attention vs. Cz alpha	0.41 (0.05–0.68)
Visual attention vs. C4 alpha	0.41 (0.07–0.67)
Visual attention vs. T4 alpha	0.42 (0.07–0.68)
Visual attention vs. O2 alpha	0.37 (0.01–0.64)
Visual attention vs. TOT beta	0.52 (0.17–0.76)
Visual attention vs. C4 beta	0.45 (0.10–0.71)
Visual attention vs. T4 beta	0.47 (0.11–0.72)
Visual attention vs. T3 beta	0.40 (0.05–0.66)
Visual attention vs. O2 beta	0.43 (0.06–0.68)
Visual attention vs. TOT beta	0.52 (0.17–0.76)
Auditory attention vs. T4 alpha	0.40 (0.04–0.66)

The same tests appear to be mainly impaired (7 children with deficits in auditory attention, 6 in visual attention).

**Fig. 2:**
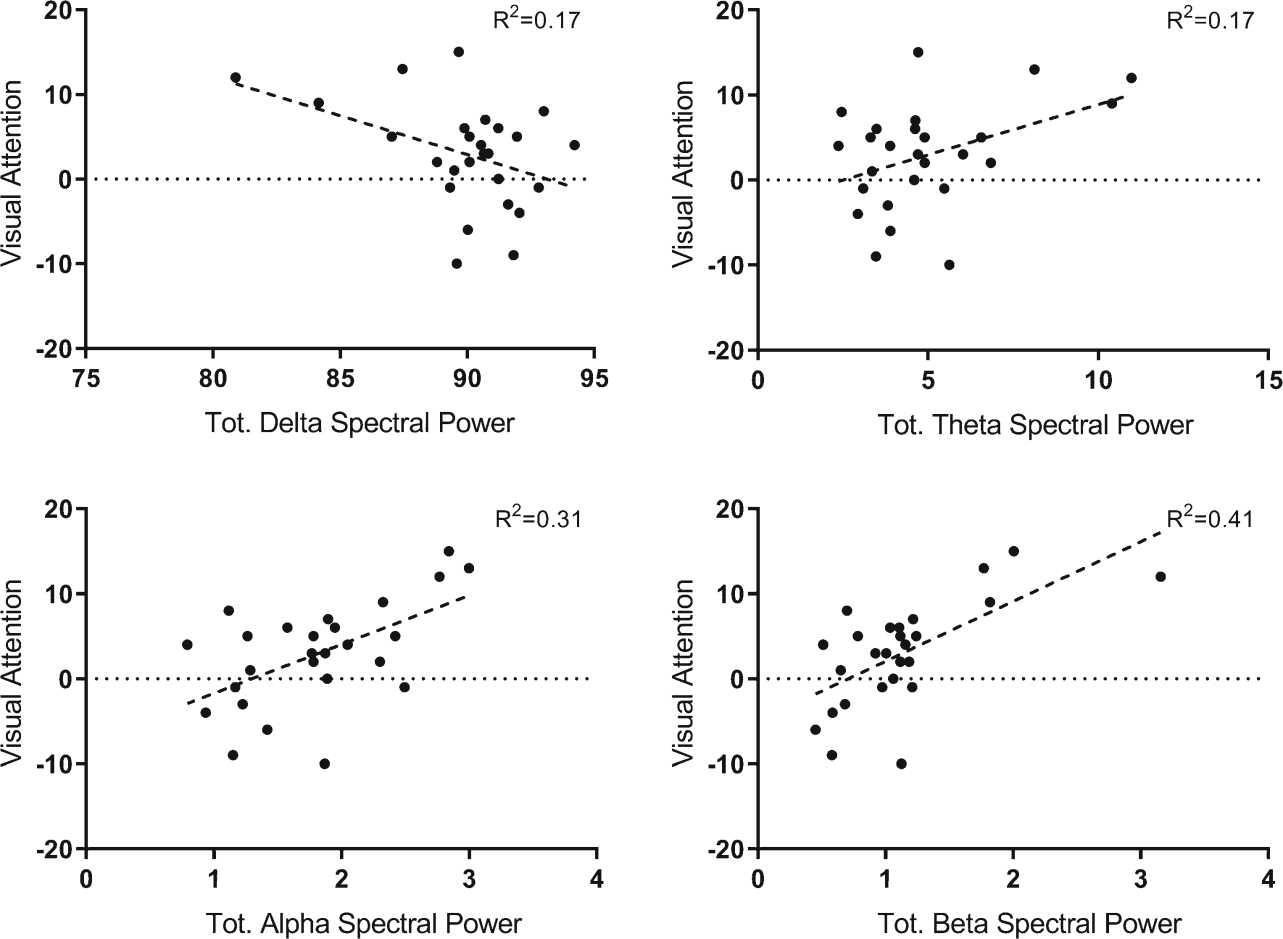
Scatterplots of performance to the visual attention task in the total delta, theta, alpha, and beta bands (raw data)

Scatterplots of performance to visual attention test and spectral values are shown in Fig. 2.

## Discussion

In the present study, we evaluated the prognostic role of spectral analysis of the EEG in those infants without medical complications. They are the most challenging group of premature infants because the prognosis is particularly tricky, and gestational age remains the unique indicator of risk.

We recorded the EEG in a crucial phase of brain development when first cortical circuitries start to develop [54]; the outcome was measured 6 years after the perinatal period, at another crucial phase of development, the school period. Outcome measures included both neuropsychological tests able to detect subtle deficits in cognition and parent’s questionnaire on child behavior.

We found that spectral EEG frequencies are independent predictors of performance in attention tasks, both in the visual and the auditory modality. In contrast, we did not found any correlations with other tasks or questionnaires and, interestingly, with gestational age.

Neonatal EEG of children performing worse to attention tasks had a relatively lower amount of power in the alpha and beta bands.

In the immature brain, the slow activity (i.e., in delta range) is the predominant feature of the background EEG [30]. It has an established role in the functional and structural shaping of neuronal circuitries [55]. By contrast, the higher frequencies are physiologically underrepresented before the beginning of the cerebral cortex maturation and progressively emerge during the last trimester of pregnancy, around 34–35 weeks of gestation [56, 57]. In fact, thirty-five-week gestation is a critical time of brain maturation: EEG background activity becomes continuous, cortical evoked potentials change from prevalent negative to positive polarity, and spectral power analysis shows increased high-frequency content [54]. These changes are due to the major development of cerebral pathways and transient organization of both neuronal circuitry and fetal brain lamination [33].

Evaluation of the emergence and characterization of spectral EEG components and their deviation from the expected typical trajectory may be important to understand early abnormalities of brain development. In a previous study, we showed, at 35 weeks post-conception, a preponderant slow and high-voltage activity in premature infants born at extremely low gestational age [28]. We speculated that an imbalance between low- and high-frequency EEG content could reflect a failure of the early developmental trajectory of the cerebral organization since it was associated with worse neurodevelopmental scores at 1 year. With the present study, we prolonged the follow-up until 6 years of age on a broader population, and we used more comprehensive and sophisticated outcome measures. We found an association between spectral EEG data and attention performance, suggesting the possibility that spectral characteristics could reflect the activity of early circuitries in the arousal–attentional system, with cascade consequences and persisting effects on the development of attention skills. Our findings may be explained by a failure in the activation of the immature cerebral cortex, reflected by the low content of high-frequency rhythms, from the ascending reticular formation, and the consequent failure of its modulatory activity [58, 59]. Research on reticular formation’s ascending pathways has demonstrated a gating activity, which enables selective attention [60, 61] and regulates gaze control as a response to arousing stimulation [62]. Therefore, the reticular activating system is implicated in the regulation of sleep-wake states and the arousal and attention systems. Studies in children born preterm support our hypothesis, reporting sleep-wake dysregulation and difficulties in sustaining and modulating attention and arousal [63, 64], orienting behavior [65], alerting [65, 66], and in tasks involving more complex attentional processes such as shifting and divided attention [67–69]. During their permanence in neonatal intensive care units, preterm infants may undergo excessive stimulation from the extra-uterine environment, despite advancements in neonatal care. It is thought that intense and unexpected stimulation such as lights, sounds, smells, and pressure signals incurred at this immature phase of maturation may compromise early formation of the arousal–attention system [70].

The impact of uncomplicated prematurity on cognition may remain latent for several years until more complex functions fail to emergence revealing the underlying neurobiological vulnerability. Complex neuropsychological functions are each other strictly interrelated. The maturation of attention is a prerequisite for the rise of the highest functions, such as flexibility, planning, and inhibition. The attention system allows better coordination of different executive components with an increase of vigilance and sustained attention [71]. After 5 years, the amount and complexity of executive skills increase dramatically. In the life of each person, executive functions have a crucial role in adaptive functioning, with consequent effects on quality of life, and recent research indicated executive dysfunctions as the core problem of some psychiatric conditions [72–75]. Furthermore, executive deficits are a frequent report after premature birth [76].

Our findings should be interpreted in light of potential limiting factors. First, the sample size is very small; this long-term longitudinal study requests the need for covering a long period (from the neonatal period to the age when complex cognitive functions develop and can be tested). In 6 years, some patients dropped out or became untraceable. However, given the high effort in the recruitment procedure, the percentage of parents who refused to participate at this stage of the follow-up was very low. Therefore, the majority of lost participants are due to logistic reasons and not the choice of parents, often biased by the effective outcome of their child. Finally, our statistical approach is reliable even with small sample sizes, but, without other confirmatory studies, the small number of patients could limit the generalizability of our results.

Another limit is due to the high number of potential confounding variables that would interact and influence the outcome in the 6 years of the life of the child. For example, we could have investigated parental mental state, known to potentially bias assessment of children’s health.

Finally, we selected a group of patients with no evident neurological risk factors other than the prematurity itself. Therefore, our results cannot be generalized to the entire population of preterm infants. However, we were specifically interested in these children in whom the outcome is highly uncertain. In premature infants with evident signs of neurological dysfunctions, the prognosis is relatively simpler. Visual inspections of EEG, MRI, and clinical evaluation may help clinicians in the diagnosis.

## Conclusion

In conclusion, the cumulative effect on the ongoing development of early disruption in cerebral circuitries [77] prompts to early identification of children at risk; results of the present study point to a possible prognostic role of the neonatal EEG spectral analysis also in the challenging group of premature infants in whom the prognosis is particularly difficult because of the absence of overt brain damage. During the time elapsed between the insult and disclosure of impairments, the developmental window for therapeutic interventions may be lost. In early infancy, rehabilitation programs can still favor changes in brain circuitry and on cortical refinement. Currently, it is important to support the achievement of the milestones at the bottom of the subsequent maturation of more complex cognitive abilities. In the absence of interventions, disrupted cerebral circuitries may accumulate during ongoing development, with deleterious cascade effects on subsequent cognitive functioning.

Future research should explore the utility of spectral EEG also in premature neonates with neurological and medical complications.

## Electronic supplementary material

ESM 1 (PDF 72 kb)

ESM 2 (DOC 137 kb)

## Abbreviations

ADHD: Attention/hyperactivity disorder
CGI: Conners global index
CRS-R: Conners’ Rating Scales-Revised
EEG: Electroencephalogram
HMC: Hamiltonian Monte Carlo
VMI: Visual-motor integration
WPPSI-III: Wechsler Preschool and Primary Scale of Intelligence III
WISC-IV: Wechsler Intelligence Scale for Children IV
