# Supplementary material for: Neonatal spectral EEG is prognostic of cognitive abilities at school age in premature infants without overt brain damage
Source: Eur J Pediatr. 2020 Sep 29;180(3):909–18. doi: 10.1007/s00431-020-03818-x (PMC7886838; doi:10.1007/s00431-020-03818-x)
Supplement: Supplementary file 1 — (PDF 72 kb) [file 431_2020_3818_MOESM1_ESM.pdf]

Cainelli et al. - EEG and Preterms

Da import and cleaning (not shown)

This code was inspired by the blog post of A. Solomon Kurz that can be found here: https://solomonkurz.netlify.com/post/bayesian-robust-correlations-with-brms-and-why-you-should-love-student-s-t/ (https://solomonkurz.netlify.com/post/bayesian-robust-correlations-with-brms-and-why-you-should-love-student-s-t/)

```
library(tidyverse)
library(readxl) #data import
library(janitor) # var names cleaning

library(brms)
library(tidybayes) #to extract 95%CI
library(rstan)
library(future) #parallelization to improve speed
```

Correlations

Correlations (n = 1508). Watch out that the file will be very large (around 5GB) so make sure to have available resources. If limited resources are available, consider to split the computation in 3 chunks (as we did and worked flawless also on a laptop). Computation time on a i5-7gen @2.4 GhZ (32GB of RAM, Windows 10) will take about 48-72 hours depending on the system configuration and priors selection.

For the explanation of `lkj = 4` see https://bookdown.org/content/1850/adventures-in-covariance.html (https://bookdown.org/content/1850/adventures-in-covariance.html)

```
neuro <- db[, 4:47] %>% #the first 3 columns were ID and date of birth
  names()

eeg <- db[, 48:83] %>%
  names()


df <- expand.grid(neuro, eeg)%>%
  as_tibble() %>%
  rename(x = Var1,
         y = Var2)

correlations <- map2(.x = df$x, .y = df$y, ~{

    model_formula <- paste("mvbind(", .x , ", ", .y, ") ~ 1") %>% as.formula()

    fit <- brm(

      data = db,
      family = student,  #this is a key point. Student, not Gaussian (default)
      model_formula,
      prior = c(prior(gamma(2, 0.1), class = nu),
                prior(normal(0, 100), class = Intercept),
                set_prior("normal(0, 100)", class = "sigma", resp = .x),
                set_prior("normal(0, 100)", class = "sigma", resp = .y),
                prior(lkj(4), class = rescor)),  # lkj = 4 permits a prior distribution that
 limit FDR
      iter = 2000, warmup = 500, chains = 4, future = getOption("future", TRUE),
      seed = 1234)

     message(glue::glue("{.x} x {.y} is finished!"))

     fit
  }

  ) %>% set_names(., nm = paste(df$x, df$y))

saveRDS(correlations, here::here("correlations.rds"))
```

95%CI extraction from models

```
intervals_df <- imap_dfr(.x = correlations, ~{

  .x %>%
    spread_draws(`rescor_.*`, regex = TRUE) %>%
    median_qi() %>%
    mutate(variables = .y) %>%
    rename_at(vars(contains("rescor")), ~ "corr")
})

saveRDS(intervals_df, here::here("intervals.rds"))
```
